# Correction: Water-floating nanohybrid films of layered titanate–graphene for sanitization of algae without secondary pollution

**DOI:** 10.1039/c8ra90039f

**Published:** 2018-05-17

**Authors:** In Young Kim, Jang Mee Lee, Eui-Ho Hwang, Yi-Rong Pei, Woo-Bin Jin, Jin-Ho Choy, Seong-Ju Hwang

**Affiliations:** Department of Chemistry and Nanoscience, College of Natural Sciences, Ewha Womans University Seoul 03760 Korea hwangsju@ewha.ac.kr +82-2-3277-3419 +82-2-3277-4370; Hwan-Il High School Seoul 04500 Korea

## Abstract

Correction for ‘Water-floating nanohybrid films of layered titanate–graphene for sanitization of algae without secondary pollution’ by In Young Kim *et al.*, *RSC Adv.*, 2016, **6**, 98528–98535.

In the published paper, the affiliations of Eui-Ho Hwang and Woo-Bin Jin were wrongly designated, and the corrected version is shown below.

The Royal Society of Chemistry apologises for these errors and any consequent inconvenience to authors and readers.

## Supplementary Material

